# Bomidin: An Optimized Antimicrobial Peptide With Broad Antiviral Activity Against Enveloped Viruses

**DOI:** 10.3389/fimmu.2022.851642

**Published:** 2022-05-19

**Authors:** Rongrong Liu, Ziyu Liu, Haoran Peng, Yunhua Lv, Yunan Feng, Junjun Kang, Naining Lu, Ruixue Ma, Shiyuan Hou, Wenjie Sun, Qikang Ying, Fang Wang, Qikang Gao, Ping Zhao, Cheng Zhu, Yixing Wang, Xingan Wu

**Affiliations:** ^1^ Department of Microbiology, School of Basic Medicine, Fourth Military Medical University, Xi’an, China; ^2^ Department of Microbiology, Second Military Medical University, Shanghai, China; ^3^ Department of Neurobiology, School of Basic Medicine, Fourth Military Medical University, Xi’an, China; ^4^ Analysis Center of Agrobiology and Environmental Sciences, Zhejiang University, Hangzhou, China; ^5^ Tianjin Key Laboratory of Function and Application of Biological Macromolecular Structures, School of Life Sciences, Tianjin University, Tianjin, China; ^6^ Jiangsu Genloci Biotech Inc., Nanjing, China

**Keywords:** antimicrobial peptide, AVP, SARS-CoV-2, HSV-2, DENV-2, CHIKV

## Abstract

The rapid evolution of highly infectious pathogens is a major threat to global public health. In the front line of defense against bacteria, fungi, and viruses, antimicrobial peptides (AMPs) are naturally produced by all living organisms and offer new possibilities for next-generation antibiotic development. However, the low yields and difficulties in the extraction and purification of AMPs have hindered their industry and scientific research applications. To overcome these barriers, we enabled high expression of bomidin, a commercial recombinant AMP based upon bovine myeloid antimicrobial peptide-27. This novel AMP, which can be expressed in Escherichia coli by adding methionine to the bomidin sequence, can be produced in bulk and is more biologically active than chemically synthesized AMPs. We verified the function of bomidin against a variety of bacteria and enveloped viruses, including severe acute respiratory syndrome coronavirus-2 (SARS-CoV-2), herpes simplex virus (HSV), dengue virus (DENV), and chikungunya virus (CHIKV). Furthermore, based on the molecular modeling of bomidin and membrane lipids, we elucidated the possible mechanism by which bomidin disrupts bacterial and viral membranes. Thus, we obtained a novel AMP with an optimized, efficient heterologous expression system for potential therapeutic application against a wide range of life-threatening pathogens.

## Introduction

The Coronavirus disease 2019 (COVID-19) pandemic has led to high mortality and new infections in many countries (1). Recently, the emergence or re-emergence of multiple viruses (severe acute respiratory syndrome coronavirus-2 [SARS-CoV-2], herpes simplex virus [HSV], Dengue virus [DENV], chikungunya virus [CHIKV], etc.), as well as drug-resistant pathogenic strains, has constantly threatened the lives of humans and animals (2). To overcome the immediate threat posed by constantly evolving pathogens and prevent future epidemics (3), broad-spectrum antimicrobial agents and novel drugs against drug-resistant strains are urgently needed (4). Antimicrobial peptides (AMPs) are amphiphilic peptides that selectively target and eliminate various microbial pathogens at low micromolar concentrations (5, 6), with little or no induction of drug resistance (7) (8). AMPs are classified into synthetic peptides and natural antibiotics. In mammals, cathelicidins and defensins constitute two prominent AMP families (9). Notably, AMPs potentially possess antiviral abilities through physical interactions with membrane or fusion proteins (10), alternation of virion morphology (11), and inducing virion aggregations (11). For example, LL-37, protegrin-1, and indolizidine have been reported to inhibit HIV infections (12). Furthermore, AMPs have attracted considerable attention as therapeutics against infectious viral pathogens such as HSV (13), DENV, Zika virus (ZIKV), Ebola virus (EBOV) (14), CHIKV (15), and, recently, SARS-CoV-2 (16–18).

Primary HSV infection usually causes a self-limited oral labia infection (HSV-1) or genital infection and subsequent persistence of latent HSV in nerve root ganglia (HSV-2) (19). Moreover, the outbreak of HSV infection may be spontaneous or induced by external stimuli such as emotional stress, ultraviolet radiation, or immunosuppression. HSV-2 infections affect more than one-half of the world’s population and can persist in sacral ganglia or trigeminal nerves with intermittent reappearance (20). Dengue hemorrhagic fever (DHF) and dengue shock syndrome (DSS) are caused by DENV-1, DENV-2, DENV-3, and DENV-4 (21). DENV-2 is the predominant cause of outbreaks (22). CHIKV is a re-emerging human arthropod-transmitted virus that may also lead to global outbreaks. It has become a serious health problem due to the lack of an antiviral treatment/vaccine. In addition, CHIKV can cause self-healing febrile diseases with joint pain. Extensive studies have been conducted to target different proteins in CHIKV to curb the spread of the virus (15).

More than 60 approved peptide drugs are currently available for sale in the United States, Europe, Japan, and Asian countries. In addition, peptide-based antiviral therapies have been approved for HIV, influenza virus, and Hepatitis virus (B and C) (23). In this new generation of compounds, AMP has antiviral activity, and its mechanism and biological properties have attracted people’s attention. The current antimicrobial peptides (APD) database is dedicated to natural antimicrobial peptides, with 190 peptides annotated as antiviral. Among these AVPs, there are 138 animals, 30 plants, 2 fungi, 1 protozoa, 14 bacteria, and 5 synthetic peptides. In addition, four famous AVP families are isolated from different natural sources-antimicrobial peptides, cecropin, cyclopeptides, and defensins (24). Applying AH-D antiviral peptides in early pregnancy can prevent Zika virus replication and offspring death (25). In addition, the peptide crossed the blood-brain barrier to reduce viral loads and damage caused by ZIKV (26). In studying the membrane binding of HCV non-structural 5A (NS5A) protein, the development of 27-polymer AH peptide preferentially ruptures lipid vesicles less than ~ 100nm in diameter. In addition to the AH peptide, the 18-mer amphiphilic α-helix C5A peptide was initially identified when screening a peptide library from HCV polyproteins and effectively inhibited various envelope viruses *in vitro*, including HCV, HIV, DENV, West Nile virus, HSV, RSV, and MV. These characteristics led to the design of C5A variants with improved antiviral selectivity and applicability for nanoparticles (27).

Because of its broad-spectrum mechanism and huge potential sequence diversity, peptides that inhibit viral entry may potentially meet the demand for new antiviral therapy; however, to optimize or evolve sequence design to combat a wide range of viral diseases, their mechanisms need to be better understood (28). Notably, the low yields and difficulties in the extraction and purification of AMPs have hindered their industry and scientific research applications. To our knowledge, only polylysine and nisin (29) can be produced industrially (29). Therefore, we developed an optimized heterologous expression system to increase AMP yields significantly, achieve efficient purification and structural AMP optimization, and improve AMP therapeutic activity (30).

The amphiphilic nature of most AMPs determines their structural flexibility. Based on their secondary structures, AMPs are usually classified into four categories: linear α-helical peptides, β-sheet peptides, β-hairpin, or loop peptides (29). Notably, the helical peptide shows an unstructured conformation in an aqueous solution, but when the peptide contacts a biological membrane, it becomes an amphiphilic spiral structure (29). BMAP-27 is a well-known peptide derived from bovine sources with a cationic NH_2_ terminus that forms an amphipathic α-helix (31). BMAP-18 is a truncated form of BMAP-27. Its toxicity in mammalian and insect cells is reduced, but it is still directly toxic to parasites *in vitro (* 32). Bomidin is BMAP-18 with a methionine added to the N-terminus of the primary sequence, which enables its expression in *E. coli*.

Bomidin (CAS#: 2374916-29-5) is a biologically extracted polypeptide containing 19 L-amino acids and with a molecular weight of 2474 Da. In the current study, we optimized the codon of the bomidin gene, induced the expression of bomidin in *Escherichia coli* BL21(DE3), and ultimately extracted and purified bomidin with purity ≥95% by ion-exchange chromatography and reversed-phase chromatography. Bomidin has shown a significant antibacterial effect against *Vibrio parahaemolyticus* and, therefore, has recognized therapeutic potential against *Macrobrachium rosenbergii* in aquaculture applications (33). To investigate the possibility of bomidin as a novel antibacterial and antiviral agent, we characterized the ability of bomidin to inhibit multiple bacterial strains and enveloped viruses (SARS-CoV-2, HSV-2, DENV-2, and CHIKV). In addition, we demonstrated the mechanism by which bomidin destroys bacterial and viral membrane structures. Our results suggest that bomidin can be further developed as a safe and efficient peptide-based therapy for treating and preventing a wide range of infections.

## Methods

### Cells, Viruses, and AMPs

Vero-E6 (African green monkey kidney cell line) and Huh7 (hepatocellular carcinoma cell line) were cultured in DMEM (Thermo Fisher Scientific, Waltham, USA) containing 10% fetal bovine serum in a 5% CO2 incubator at 37°C. DENV-2, HSV-2, and HTNV were kept in our lab and propagated in Huh7 and Vero-E6 cells. The SARS-CoV-2 virus (PubMed No: MT627325) was isolated, processed, and maintained in the ABSL-3 laboratory. In addition, CHIKV and SARS-CoV-2 were gifts from the Second Military Medical University and propagated in Vero-E6 cells. Detection of virus titers was performed using plaque-formation assays (SARS-CoV-2, HSV-2, and CHIKV), fluorescent focus assays (DENV2), and ELISAs (HTNV). Bomidin: MGRFKRFRKKFKKLFKKLS (CAS#: 2374916-29-5) was provided by Genloci Biotech with purity > 95%. (Nanjing, China). In addition, BMAP-18 (GRFKRFRKKFKKLFKKIS) (34) and RI-10 (RIVQRIKDFL) (14) control peptides were synthesized by GenScript (Genscript Biotech Corporation China). All peptide AMPs were dissolved in ddH_2_O at a dose of 1 mM and diluted in the medium as required before the assay.

### Character Determination of Bomidin

The molecular weight of Bomidin was obtained using an ultra-extreme mass spectrometer (Bruker Daltonik GmbH, Leipzig, Germany). The data are collected for a mass range from m/z 500–3000 Da in positive reflection mode. At a laser frequency of 1000 Hz, 1000 excitations are obtained at each point.

### Cytotoxicity Measurement

The cell suspension (2×10^4^ cells/well) was inoculated in a 96-well culture plate and grown overnight to 90% confluence at 37°C. The cells were treated with different concentrations of AMPs for 1 hour, and the same concentration of PBS was used as a control. After 48 hours of cell culture, 10% CCK8 solution was added to each well and incubated at 37°C for 1 hour. The absorbance was measured at 450 nm using a microplate reader. The CC50 represents the drug concentration required for the uninfected cells’ 50% cell killing (cytotoxicity).

### *In Vitro* Antibacterial Assays

The test strain’s minimal inhibitory concentration (MIC) was determined by the microbroth dilution method recommended by the Clinical and Laboratory Standards Institute (CLSI) M07-A11. The MIC of compounds to fungi was determined by the micro liquid-based dilution method recommended by [Reference Method for Broth Dilution Antifungal Susceptibility Testing of Yeast; Approved Standard-third Edition (Vol, 28, No. l4); M27-A3]. The MIC assay was performed according to the method described in the **Supplementary Materials**.

### Fluorescent Focus Assay

Huh, 7 cells were seeded in 12-well plates and incubated under 5% CO2 at 37°C overnight. Viruses were mixed with different concentrations of Bomidin (0 μM, 10 μM, 20 μM, 40 μM, and 80 μM) for 30 min and infected the cells for 2 hours. After adsorption, an overlay of DMEM, 5% FBS, and 0.8% carboxymethylcellulose (CMC, ICN Biomedicals, Aurora, OH) was added. The infected monolayer cells were cultured in a 5% CO2 incubator at 37°C. The covering solution of the culture medium infected for 2 days was removed, and cold phosphate-buffered saline (PBS) was added. After incubating for 5 minutes, the PBS was discarded, and the cells were fixed with cold absolute methanol (Sigma–Aldrich Co., St. Louis, MO) for 10 minutes and then washed with PBS. Next, the cells were incubated with an antibody diluted with PBS for 1 hour at 37°C, washed three times, and then stained with a secondary antibody. The fluorescent foci of infection were observed and counted by fluorescence microscopy.

### Immunofluorescence Assay

Different viruses were mixed with antimicrobial peptides for 10 min, infected cells for 1 h, and cultured with DMEM containing 10% serum for 48 h. After discarding the supernatant and washing it three times with PBS, it was permeated with PBS containing 0.2% Triton X-100 (containing 0.1% BSA) at room temperature for 30 minutes. Then, the cells were mixed with an antiviral antibody at 4°C overnight. After washing with PBS three times, the cells were incubated with fluorescence-conjugated secondary antibodies at room temperature for 1 hour. Cell nuclei were stained using 4’,6’-diamidino-2-phenylindole (DAPI) in the dark for 5 min, and images were obtained by Fluorescence microscope.

### Plaque Forming Assay

The cells were cultured overnight in a 6-well plate (1×106 cells/well). Two hours after adding viruses to the wells, the cells were further incubated with a maintenance medium containing 2% methylcellulose (Sigma, Saint Louis, USA) for 48 hours at 37°C. After incubation, the maintenance medium was removed, and the cells were washed with phosphate-buffered saline (PBS) and stained with methylene blue.

### Quantitative Real-Time PCR Assay

Cellular RNA was isolated using a standard protocol, and qRT–PCR analysis was performed using SYBR Green (BIO-RAD, California, USA) according to the manufacturer’s protocol. Viral RNA expression was calculated using the 2-delta delta C.T. (cycle threshold) method normalized to GAPDH expression. qPCR primers sequences are as follows: SARS-CoV-2: Fwd-5’GGGGAACTTCTCCTGCTAGAAT 3’, Rev 5’CAGACATTTTGCTCTCAAGCTG 3’ | HSV-2: Fwd-5’ AATGTGGTTTAGCTCCCGCA-3’, Rev-5’ CCAGTTGGCGTGTCTGTTTC-3’ | DENV-2: Fwd-5’ GGTTTTGGGAGCTGGTTGAC, Rev-5’ ACTCTAAGAAGCGTGCTCCA |CHIKV:Fwd-5’TCTATAACATGGACTACCCGCCC, Rev-5’ AGCCAGATGGTGCCTGAGAGT

### Western Blotting

The cells were washed twice with DPBS, lysed with 1×SDS protein loading buffer at 4°C for 30 minutes, and harvested. The supernatant was collected by centrifugation (13,000 rpm) at 4°C for 30 minutes. Next, the same amount of protein was boiled at 95°C for 10 minutes, separated by SDS–PAGE with different concentrations, and then transferred to a polyvinylidene fluoride membrane by electrophoresis. After blocking with 5% skim milk in 1×TBS, the membrane was incubated with primary antibody (Abs), and then the secondary Abs were labeled with infrared dye. The Odyssey infrared imaging system (LI-COR Biosciences) was used to visualize the signal on the polyvinylidene fluoride film and then the whole W.B. program.

### Transmission Electron Microscopy

After predehydration with graded ethanol, the bacteria were transferred to anhydrous acetone for 20 minutes. At room temperature, the sample was placed in a 1:1 mixture of absolute acetone and final Spoor resin mixture for 1 hour and then transferred to a 1:3 mixture of absolute acetone and final resin mixture for 3 hours. The final Spoor resin mixture was incubated overnight. Next, the sample was placed in an Eppendorf tube containing Spoor resin and heated at 70°C for more than 9 hours. Samples were sliced in a Leica EMUC7 ultramome, and the slices were stained with uranyl acetate and essential lead citrate for 5 to 10 minutes and observed with a Hitachi H-7650TEM.

Virus-infected cells were collected by centrifugation to prepare cell particles, fixed in cacodylate sodium buffer containing 1% glutaraldehyde (0.2 M, pH 7.2), and fixed in 1% osmium tetroxide. Then, the fixed sample was dehydrated by acetone solution and embedded in epoxy resin. Finally, the sample was polymerized at 60°C for 3 days. The resin block was used to prepare ultrathin slices (50-70 nm thick). These sections were supported by copper mesh, negatively stained with uranyl acetate and lead citrate (electron microscopy) and observed with a JEM100SX transmission electron microscope (JEOL, Tokyo, Japan).

### Molecular Dynamics Simulations

The all-atom M.D. simulations were performed on Gromacs 2019.6 with CHARMM36 force field (35, 36). The initial model of the bomidin peptide and various lipids, along with 150 mM NaCl and explicit water molecules, were constructed by CHARMM-GUI and equilibrated as an NPT ensemble at 303.15 K (37, 38). The simulations were carried out with LINCS constraints on H-bonds and a time step of 2 fs. The nonbonded interaction cutoff for electrostatic calculations was set as 10 Å, and the particle mesh Ewald (PME) method was used to calculate long-range electrostatic interactions.

### Statistical Analysis

Statistical analysis was performed by GraphPad Prism software (version 9.0, GraphPad Software Inc.). Data are expressed as the means ± S.D. Unpaired Student’s t tests determined statistical significance between the two groups. One-way ANOVA determined the significance of the variability between different groups. p < 0.05 was considered statistically significant, and p > 0.05 was considered statistically nonsignificant (*p < 0.05; **p < 0.01; ***p < 0.001; NS, no significance).

## Results

### Peptide Prediction and Characterization

**Figure 1:**
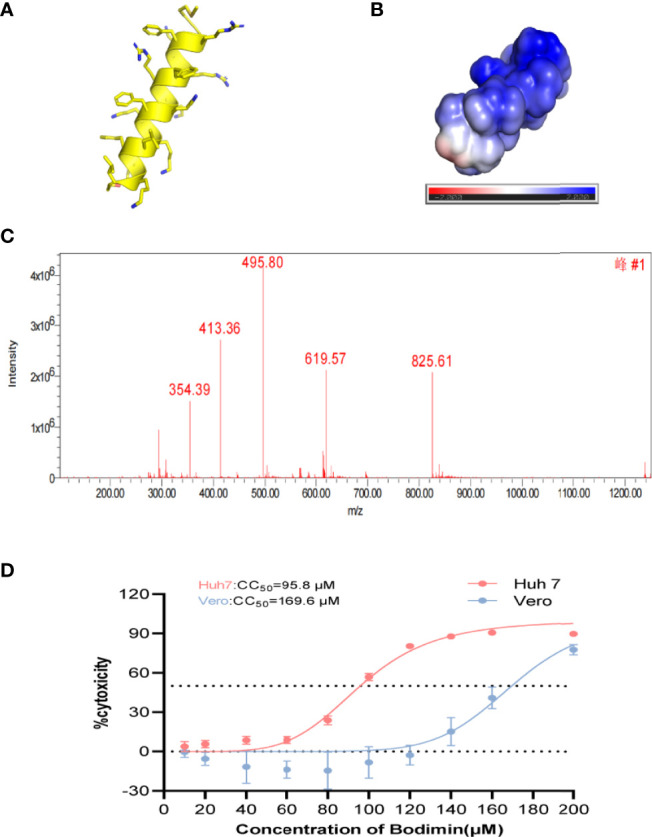
The structural characteristics of bomidin. **(A)** The α-helix of bomidin. **(B)** Total solvent accessibility of bomidin; blue, hydrophobic; red, hydrophilic regions. **(C)** Mass analysis of bomidin. **(D)** Cytotoxic effects of bomidin in cells. The cytotoxicity induced by bomidin in Vero E6 and Huh7 cells was determined by CCK-8 assay 48 h after treatment. CC50 is the bomidin concentration required to reduce cell viability by 50%.

We simulated the structure, hydrophilicity, and hydrophobicity of bomidin, which is an αα-helical peptide (**Figure 1A**), with positively charged lysine and arginine residues scattered among the hydrophobic parts on both sides (**Figure 1B**). In a dissolved state, bomidin has a charge number related to the pH of the solution. The mass spectrum in **Figure 1C** shows the multiple charge states (from +7 to +3) of the bomidin ion detected. Considering that as many as 10 in the bomidin sequence as a primary amino acid, we speculate that Bomidin may also have ions of various valence states in a neutral solution (**Figure 1C**). The mass of bomidin was 2474 Da.

### Bomidin Inhibits 12 Bacterial Species and 2 Fungal Strains

AMPs featured high specificity against bacteria and relatively low cytotoxicity toward mammalian cells. Therefore, we first examined the impact of bomidin on cell viability. The 50% cytotoxicity (CC50) value in virus-susceptible cells was 169.6 ± 1.1 μM in Vero cell lines and 95.8 ± 1.1 μM in Hu7 cell lines (**Figure 1D**). We then established that bomidin inhibited 12 bacterial species, including 41 strains. To assess the effects of bomidin on common bacteria, we measured the minimum inhibitory concentration (MIC) using the microbroth dilution method (39). We determined the effects of bomidin on other bacterial strains, including 8 gram-positive and 4 gram-negative bacteria species (**Table 1**). The MICs were in the range of 1-4 μM in common. For the more tolerant gram-positive bacterium *Enterococcus faecalis* and the fungus *Candida albicans*, the MIC value ranged from 8 to 32 μM. (**Table 1**).

**Table 1:** MICs of Bomidin against various bacteria and fungi.

Organism		MIC range (μM)
Gram Positive Bacteria	*Staphylococcus aureus* (4 strains)	2∼4
	*Bacillus megaterium* Bm 11	2
	*Bacillus subtilis* KCTC 3068	4
	*Staphylococcus epidermidis* KCTC 1917	4
	*Enterococcus faecalis* (10 strains)	8∼> 32
	*Enterococcus faecium* (5 strains)	8∼> 32
	*Streptococcus agalactiae* (3 strains)	1∼4
	*Acinetobacter baumanni* (10 strains)	0.5∼16
Gram Negative Bacteria	*Escherichia coli* (3 strains)	2∼4
	*Salmonella typhimurium* ATCC 14028	4
	*Pseudomonas aeruginosa* ATCC 27853	1
	*Serratia marcescens* ATCC 8100	2
Fungi	*Candida albicans*	16
	*Cryptococcus neoformans*	4
Drug-resistant Bacteria	Vancomycin-resistant *Staphylococcus aureus*	> 50
	Extended-spectrum β-lactamases (ESBLs)-producing *Escherichia coli*	> 50
	Multiple drug-resistant *Pseudomonas aeruginosa*	> 50
	Multiple drug-resistant *Acinetobacter baumanni*	> 50
	Multiple drug-resistant *Klebsiella*	50

The effects of Bomidin on other bacterial strains, including 8 Gram-positive bacteria and 4 Gram-negative bacteria, and the MICs were in the range of 1-4 μM. For the more tolerant Gram-positive bacteria Enterococcus faecalis and the fungi Candida albicans, the MIC value ranges from 8-32μM. For the drug-resistant bacteria, the MIC value is greater than or equal to 50μM.

**Figure 2:**
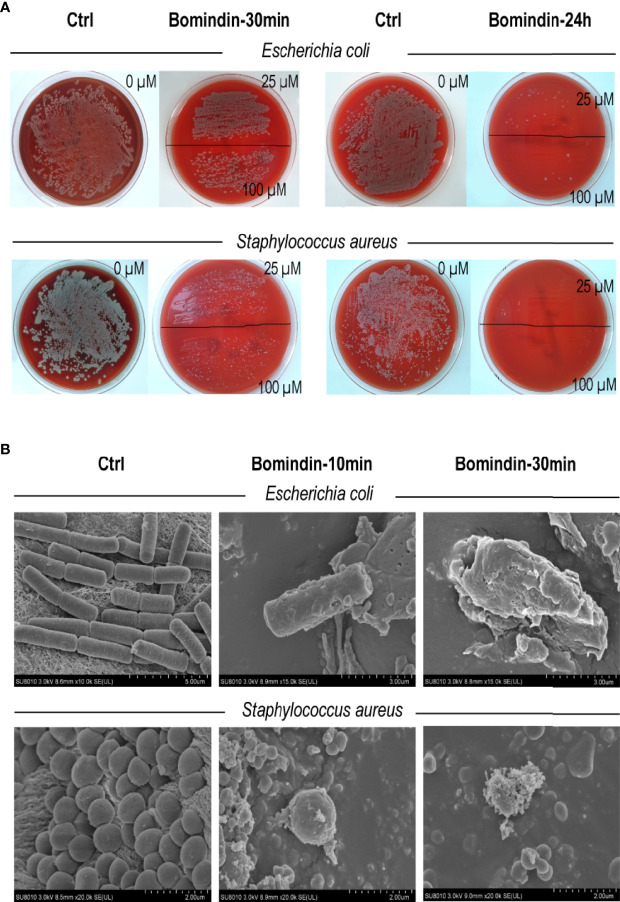
Assessment of bomidin as an inhibitor of bacterial growth. **(A)** The 25 μM and 100 μM doses of bomidin treatment significantly reduced bacterial populations within 30 min while abolishing the growth of ESBL-producing *Escherichia coli* and *Staphylococcus aureus* within 24 h. **(B)** After 30 min or 1 h of bomidin treatment, the number and size of bacteria (*B. subtilis, E coli*, and *S. aureus*) decreased. Perforations, vesicular structures, and some flocculent material aggregates were observed on the membranes, and some bacteria ruptured into fragmented, unclear structures.

Specifically, we characterized the antimicrobial effects of bomidin on 5 common pathogenic drug-resistant strains from hospitals. These drug-resistant strains (extended-spectrum β-lactamase (ESBL)-producing *E. coli*, vancomycin-resistant *Staphylococcus aureus*, multidrug-resistant *Acinetobacter baumannii, Klebsiella* and *Pseudomonas aeruginosa*) were cultured on blood agar plates with complete hemolytic rings (*β*-hemolytic phenotype). Treatment with bomidin significantly reduced the populations of these pathogens. At a 100 μM dose, bomidin abolished the growth of *Klebsiella* and *P. aeruginosa* within 30 minutes, while its effects on the cultures of *S. aureus, A. baumannii*, and ESBL*-*producing *E. coli* were evident within 24 h (**Figure 2A** and **Supplementary Figure 1**).

We then used a scanning electron microscope to directly observe the bomidin-treated bacterial samples. The morphology of the untreated cells was intact (**Figure 2B**, left column). In contrast, the membrane surface of the bomidin-treated cells was swollen, the cell surface was rough, and the morphology showed changes (**Figure 2B**). After 10 min or 30 min of bomidin treatment, the number of bacteria (*B. subtilis, E. coli*, and *S. aureus*) decreased, and their size also reduced. In addition, perforations, vesicular structures, and flocculent material aggregates were observed on the membranes, and some bacteria had ruptured, presenting fragmented, unclear structures (**Figure 2B**). Hence, bomidin disrupted the integrity of the bacterial membranes.

### Bomidin Inhibits a Broad Range of Enveloped Viruses at the mRNA and Protein Levels

**Figure 3:**
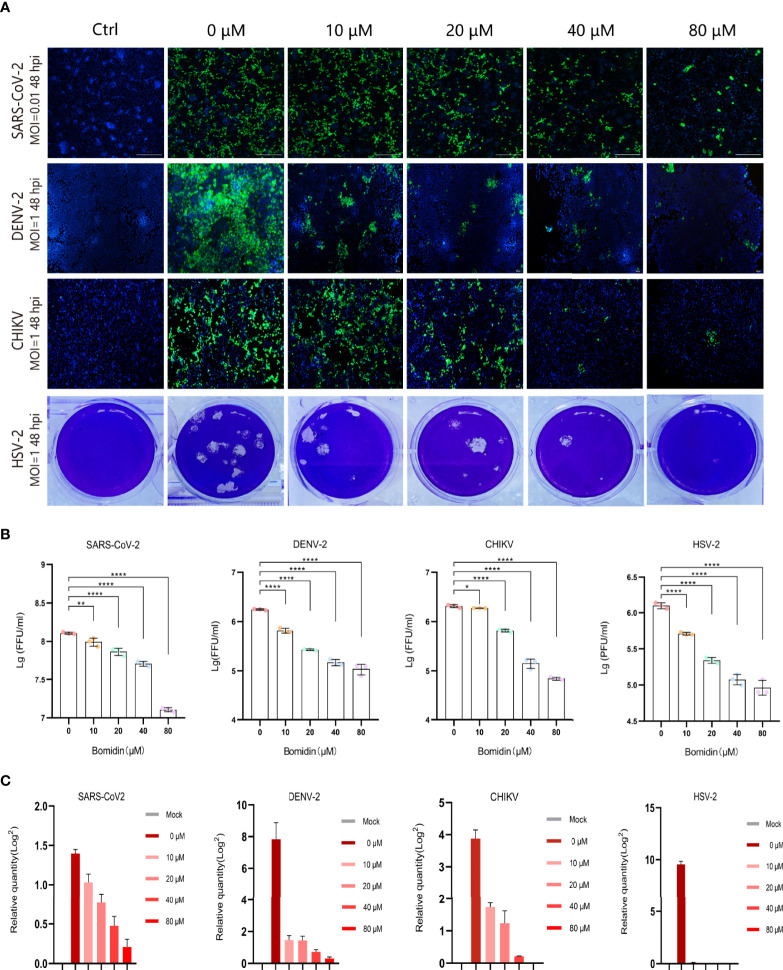
Broad spectrum antiviral activity of bomidin against viral infection in cells. **(A)** The dose-dependent inhibitory effect of bomidin at different concentrations (0 μM, 10 μM, 20 μM, 40 μM and 80 μM) was determined by an immunofluorescence assay (SARS-CoV-2, DENV-2 and CHIKV) and a plaque forming assay (HSV-2) 48 h postinfection. Cell nuclei were stained with DAPI (blue). The viral proteins are indicated in green. **(B)** Fluorescence quantification. **(C)** Bomidin inhibition of virus RNA formation at different concentrations (0 μM, 10 μM, 20 μM, 40 μM and 80 μM) was determined by quantitative real-time PCR 48 h postinfection, and GAPDH was used as the housekeeping gene for normalization. The data are shown as the means ± S.D.; n = 3 cell cultures per experiment. *p < 0.05; **p < 0.01; ****p < 0.0001.

As enveloped viruses (SARS-CoV-2, HSV-2, CHIKV, DENV-2, etc.) contain viral membranes, we were interested in testing the antiviral effects of bomidin on these representative viruses. After incubating bomidin and the viruses for 10 minutes, we infected these susceptible cells with SARS-CoV-2, HSV-2, CHIKV, or DENV-2. We quantified the replication ability of the remaining viruses at the protein level using immunofluorescence (live SARS-CoV-2 and DENV-2) or GFP reporter genes (for pseudotyped CHIKV) (**Figures 3A, B**). Bomidin inhibited the infectability of each virus in a dose-responsive manner. Specifically, 10 μM bomidin was enough to reduce the population of DENV-2 or HSV by 50%, while 80 μM bomidin significantly inhibited SARS-CoV-2 growth. We also corroborated the inhibitory effects at the mRNA level. Consistent with the measurements of GFP signals, the DENV-2 and HSV mRNA levels were susceptible to increased bomidin concentrations (**Figure 3C**).

### Electron Microscopy Revealed That the Number of Viral Particles Decreased Significantly After Bomidin Treatment

**Figure 4:**
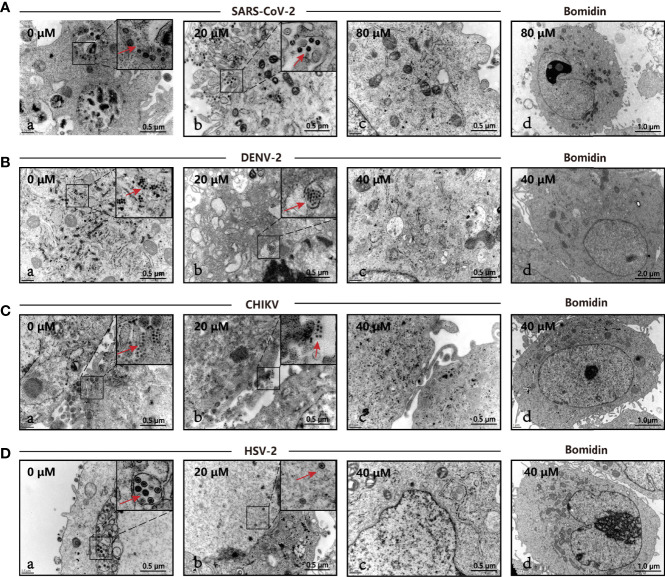
The antiviral activity of bomidin against viral infection was determined by transmission electron microscopy (TEM). The different viruses were mixed with the respective concentrations of bomidin-treated cells for 2 hours and detected by TEM 48 h postinfection. **(A)** SARS-CoV-2, **(B)** DENV-2, **(C)** CHIKV, **(D)** HSV-2. **(a)** Virus-infected cells; **(b, c)** Bomidin-treated viral cultures for 10-30 min at 37°C with infected cells; red arrows: viral particles. **(d)** Bomidin control.

We directly compared viral particles in cells with or without bomidin treatment using electron microscopy. In cells without bomidin, dark-colored viral particles of SARS-CoV-2, HSV-2, CHIKV, or DENV-2 were apparent. With 20µM, 40 µM or 80 µM bomidin pretreatment, no viral particles were observed, suggesting an antiviral inhibition percentage greater than 90% (**Figures 4A–D, a-c**). Bomidin did not affect the integrity of the mammalian cell membrane (**Figures 4A–D, d**).

### Compared With Other Reported Antibacterial Peptides, Including BMAP-18, Bomidin Shows a More Significant Inhibitory Effect on Virus Infection

**Figure 5:**
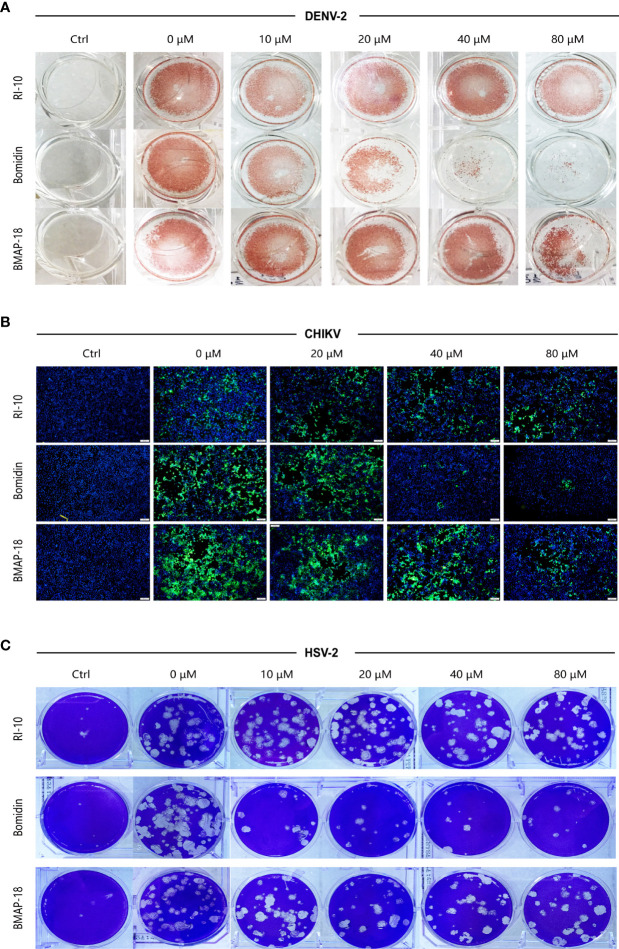
Bomidin shows better antiviral activity than the antimicrobial peptide BMAP-18.2. Comparison of the antiviral effects of the antimicrobial peptides bomidin and BMAP-18 on different viruses. The viruses were mixed with varying concentrations of the antimicrobial peptide to infect cells for 2 h. The effects were detected by various methods **(A)** fluorescence focus assay; **(B)** immunofluorescence assay; **(C)** plaque-forming assay) 48 h postinfection. RI-10: nonantimicrobial control peptide; BMAP-18: antimicrobial control peptide; bomidin: BMAP18-derived antimicrobial peptide.

We compared the antiviral activities of the mammalian cathelicidins BMAP-18 and bomidin and the control peptide RI-10 (RIVQRIKDFL) (14). As shown in spot tests, immunofluorescence assays, and plaque tests, the ability of bomidin to inhibit infection (DENV-2, CHIKV, and HSV-2) was significantly higher than that of the other two peptides (**Figures 5A–C**).

### Bomidin Can Pass Through Bacterial and Viral Membranes in Simulated Systems But Does Not Damage Eukaryotic Cell Membranes

**Figure 6:**
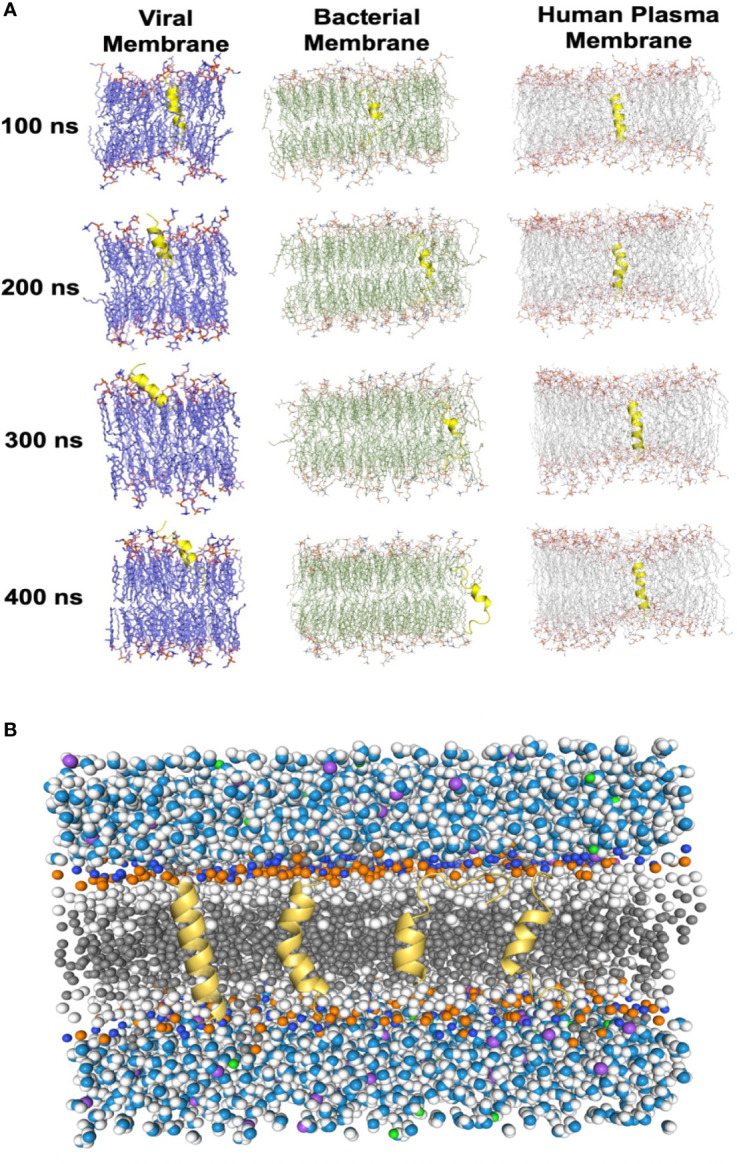
Molecular dynamics simulations of the Bomidin–membrane interactions. **(A)** Snapshots of the Bomidin and bilayer membrane dynamic systems throughout the 400 ns trajectories. Bomidin interacted strongly with anionic phospholipid bilayers with a preference for fluid layers. Membrane penetrations or disruptions were observed for viral/bacterial membrane models (left/middle panel). The elevated cholesterol-to-phospholipid molar ratio (commonly seen in mammalian plasma membranes) and decrease in membrane fluidity can shield the membrane from the action of the peptide (right panel). **(B)** Lateral view of the overall simulated system with a bilayer of lipids (dark gray) immersed in water (blue/white spheres) and ions (purple/green spheres). Conformations sampled by Bomidin (yellow) are shown.

To evaluate the impacts of the bomidin peptide on different components of cellular or viral membranes, we computationally modeled systems containing bomidin and common membrane lipids (**Figure 6** and **Supplementary Table 1**) (phosphatidylserine, P.S.; phosphatidylinositol, P.I.; phosphatidylcholine, P.C.; phosphatidylethanolamine, P.E.; cholesterol, Chol; and sphingomyelin, S.M.). In current study, the components of viral membrane were adapted from shared lipidome of DENV (40), SARS-CoV-2 (41), HSV-2 (42), E. Coli and mammalian cell membranes (43) were used as the model system. Notably, the mammalian cell plasma membrane contains significant amounts (~ 20%) of cholesterol, while a majority of viral membranes are composed of P.C. (~60%), P.E. (~20%), and P.I. (~10%) lipids. When embedded inside the bilayers of a viral or bacterial membrane model, bomidin disrupted the surrounding lipid molecules and emerged from one side of the membrane bilayer (**Figure 6A** and **Supplementary Video 1**), possibly leading to the perforations or vesicular structures observed in the electron microscopy images. In contrast, bomidin resides in the plasma membrane without disturbing its overall integrity, likely because the enriched components of cholesterol reduced the fluidity of human plasma membranes. We postulated that the efficacy of bomidin was sensitive to the chemical nature of lipids it interacted. As control simulations, we also evaluated the effects of several known AMPs with varying lengths/conformations (**Supplementary Video 2**, two antibacterial peptides and one anti-HCV peptide). The results of our control simulations indicated that the bilayer system we constructed for molecular dynamics simulations reflected the antimicrobial activities; all three peptides caused membrane permeation and disruption.

## Discussion

Collectively, our results demonstrated the potential of bomidin as a broad-spectrum inhibitor of bacteria and multiple enveloped viral infections. As a family of cationic peptides, the antimicrobial or antiviral activity is usually associated with its ability to adsorb on bacterial/viral surfaces or with interactions with innate immune system components to enhance nucleic acid-sensing (44). Specifically, AMP membrane-targeting mechanisms can be described through pore models or carpet models (45). AMPs vertically embedded in the cell membrane accumulate and then bend to form a circular hole with a 1–2 nm diameter (also known as the toroidal pore or wormhole model, **Figure 6B**) (45). Interestingly, our data indicated that Bomidin disrupted flavivirus membranes but did not affect the integrity of Hantaan virus (HTNV) membranes (**Supplementary Figure 3**) or eukaryotic cell membranes. Previous studies have suggested that flavivirus membranes are derived from internal endoplasmic reticulum membranes of infected cells (46). Hence, we postulated that the function of bomidin as an antiviral peptide depended on the lipid composition and chemical properties of the membranes with which it interacts. Indeed, molecular modeling showed that bomidin readily disrupted viral membranes (with components similar to those in the endoplasmic reticulum) and bacterial membranes but did not alter the overall structures of the cell membranes, corroborating our experimental observations.

The recent trend of virus epidemics shows that enveloped viruses are the main threat to global health. Therefore, it will be beneficial to formulate a widely applicable anti-virus strategy for a wide range of enveloped viruses. The phospholipid membrane, including the viral envelope, is a suitable target, especially if the strategy based on nanotechnology can be further optimized to target the viral membrane selectively (47). Previous studies suggested the lipid composition of the virion envelope reflects that of the membrane where budding took place (48). The Lipid Envelope Antiviral Disruption (LEAD) molecules (such as CLR01) were shown to broadly inhibit mosquito-borne viruses (such as DENV and ZIKV) and other lipid membrane-enveloped viruses (49). It disrupts the lipid envelope surrounding virions, abrogates viral infectivity, and reduces viral load (49).

We observed broad-spectrum inhibitory activities against over forty different bacteria and four enveloped viruses, suggesting that the bomidin peptide could target lipid components shared among the bacteria and viruses. Furthermore, based on recently published lipidome experiments of viruses, specifically DENV (40), SARS-CoV-2 (41), HSV (42), and studies of E. Coli and mammalian cell membranes (43). We constructed model systems of commonly shared lipid components from a consensus of lipidomic measurements to investigate the role of bomidin on the model membrane (48). Hence, we further divided our membrane models into those resembling the endoplasmic reticulum/Golgi or those resembling the plasma membrane. A notable difference between these two is the enrichment of cholesterol esters and sphingomyelin (50). Using these models, we could explain why bomidin readily inhibited viruses budding from the E.R./Golgi (such as Dengue and SARS-CoV-2) but was not as effective against HSV-1 (high amount of sphingomyelin) or HTNV (budding from the plasma membrane) (**Supplementary Figure 3**). The lipid components of E. Coli inner membrane and human cell plasma membrane were used as positive controls and negative controls, respectively, in our simulations (43). An emerging paradigm for broad-spectrum antimicrobial molecules focused on the lipids of the virus and bacteria (51), and our results reinforced that interfacial activity of AMPs correlated with their broad-spectrum antimicrobial activities (11).

More than 400 peptide drugs are currently in clinical trials, and the FDA has approved approximately 60 kinds of peptide drugs (23). In sharp contrast, few peptides have been approved for use in antiviral treatment. Therefore, strategies are being developed to improve drug properties, such as permeability, oral bioavailability, and blood and cell stability (29). Despite a few potential limitations (such as limited mass production), peptides exhibit advantages over small-molecule drugs (organics), including superior binding affinity and fewer side effects (14, 23, 52). Furthermore, the backbone and side chains of natural AMPs can be conveniently adapted to carry a circular structure consisting of D-amino acids (53), which significantly increases the stability of the peptides and improves their drug properties in the presence of peptidases (29).

In summary, the protective effects of bomidin reported here suggest further expansion of the antiviral arsenal. Furthermore, our rational engineering of bomidin has enabled its mass production. It generates new opportunities for future manufacturing and applications, such as loaded bomidin acting as nanocarriers to achieve sustained drug release, external medicines, nasally administered mist sprays, or wound dressings. Overall, we expect that the systemic administration and development of bomidin into antibiotics and broad-spectrum antiviral drugs will contribute to our constant fight against drug-resistant bacterial infections and viruses and stall the spread of worldwide pandemic-causing pathogens.

## Data Availability Statement

The raw data supporting the conclusions of this article will be made available by the authors, without undue reservation.

## Author Contributions

RL: Conceptualization, data curation, formal analysis, investigation, methodology, software, validation, visualization. ZL: Data curation, formal analysis, methodology, software, validation, visualization, writing—original draft, writing—review and editing. HP: Data curation, formal analysis, methodology. YL: Methodology. YF: Investigation, writing—original draft. JK: Data curation, methodology, software. NL: Data curation. RM, Methodology. SH: Methodology. WS: Methodology. QY: Methodology. FW: Methodology, supervision. QG: Conceptualization, methodology, resources. PZ: Data curation, formal analysis, resources, supervision. CZ: Data curation, formal analysis, funding acquisition, methodology, validation, visualization, writing—review and editing. YW: Conceptualization, data curation, funding acquisition, methodology, resources. XW: Funding acquisition, methodology, project administration, resources, supervision, writing—review and editing. All authors contributed to the article and approved the submitted version.

## Funding

This work was supported by the National Natural Science Foundation of China (Nos. 81772167, 81971563 to XW), the Key Research and Development Project of Shaanxi Province (No. 2019ZDLSF02-03 to XW), the National Key Research and Development Program of China (2020YFA0908501 to CZ), the National Natural Science Foundation of China (22007071 and 22077094 to CZ), and the project of Shaanxi Social Development of Science and Technology (NO.2016SF-112 to ZL).

## Conflict of Interest

Author YW was employed by the company Jiangsu Genloci Biotech Inc.

The remaining authors declare that the research was conducted in the absence of any commercial or financial relationships that could be construed as a potential conflict of interest.

## Publisher’s Note

All claims expressed in this article are solely those of the authors and do not necessarily represent those of their affiliated organizations, or those of the publisher, the editors and the reviewers. Any product that may be evaluated in this article, or claim that may be made by its manufacturer, is not guaranteed or endorsed by the publisher.
