# Supplementary material for: Bomidin: An Optimized Antimicrobial Peptide With Broad Antiviral Activity Against Enveloped Viruses
Source: Front Immunol. 2022 May 19;13:851642. doi: 10.3389/fimmu.2022.851642 (PMC9160972; doi:10.3389/fimmu.2022.851642)
Supplement: Supplementary file 1 [file DataSheet_1.docx]

Supplementary Material

# Supplementary data.

Methods:

**2.4 In vitro antibacterial assays**

**2.4.1 Bacterial strains and their cultivation.**

The clinical isolates and standard strains screened by antibacterial activity in vitro are shown in Table 1. The following strains are all clinical isolates collected in Sichuan, Guangdong and Beijing from May 2017 to December 2019. In the collection unit, it was identified by VITEK-60 automatic microbiological identification instrument, and then re-identified by conventional methods . Each strain of bacteria was purified by single colony on Agar plate before the experiment, 37. (3) the bacteria cultured freshly overnight were diluted properly for the experiment. Quality control strain: purchased from ATCC.

**2.4.2 Media and cultivation conditions**

Staphylococcus, Enterococcus, Enterobacter: CAMHB (Cation Adjusted Mueller-Hinton) (BD) cultured medium. Streptococcus: CAMHB medium containing 2.5% to 5% horse blood. Moraxella catarrhalis: CAMHB medium. Haemophilus influenzae: HTM (Haemophilus Test Medium) (Gibco). Fungi: RPMI1640 medium (Gibco).

**2.4.3 MIC assay**

The test strain's minimal inhibitory concentration (MIC) was determined by the micro broth dilution method recommended by the Clinical and Laboratory Standards Institute (CLSI) M07-A11. The MIC of compounds to fungi was determined by micro liquid-based dilution method recommended by [Reference Method for Broth Dilution Antifungal Susceptibility Testing of Yeast; Approved Standard-third Edition (Vbl,28, No.l4); M27-A3].

Weigh an appropriate amount of sample powder (control ambient humidity less than 60%) according to the solubility of the sample, and dissolve the sample with pure, sterile water or DMSO (dimethyl inkstone) concentration of the mother liquor is 1.28mg/mL. Take an appropriate amount of mother liquid with aseptic broth and dilute it tenfold to 0.128mg/mL. Half the volume of the solution is packed into a 96-hole sampling tank, and the other half volume is diluted twice with aseptic broth and then added to the deep-hole sampling tank. Repeat the above steps so that the drug concentration in the adding tank is 128,64,32,8,4,2,0.5,0.25 &gt; 0.125, 0.06mg/L. Three replicates were used for each sample.

Several colonies were selected from Agar plates cultured for 18-24 hours to make bacterial suspension directly in sterile normal saline, and the concentration of bacterial suspension was adjusted to 0.5 McDonnell unit. Preparation of bacterial suspension: the corrected bacterial liquid was diluted to (4-8) x10^5^CFU/ml with broth, which is now used. Preparation of fungal suspension: dilute the corrected bacterial solution to (1-5) x10^3^ / ml. The sample solutions of different concentrations mentioned above were absorbed into wells 1 to 12 of the sterile 96-well polystyrene plate, respectively, and the inoculum 100ul was added to each well. The final concentrations of the tested samples in the wells were 64,32,16,8,4,2,1,0.5,0.25,0.125, 0.06 and 0.03mg/L (the test concentration range of some levofloxacin was 16-0.008mg/L). The final inoculation concentrations of bacteria and fungi were (2-4) x10^5^CFU /ml and (0.5-2.5) x10^3^ /ml, respectively. Another growth control well containing 100ul inoculum and 100ul aseptic broth was set up. The test substance was mixed with the inoculum and sealed in each well.

The 96-well plate after inoculation was incubated at 37°C, and the bacteria needed to be set for 20 hours. The fungi need to be incubated for 48 hours. At the end of the culture, the growth of bacteria in each pore was observed. The lowest drug concentration that completely inhibited bacteria's growth in the well was taken as the MIC.

# Supplementary Figures and Tables

##
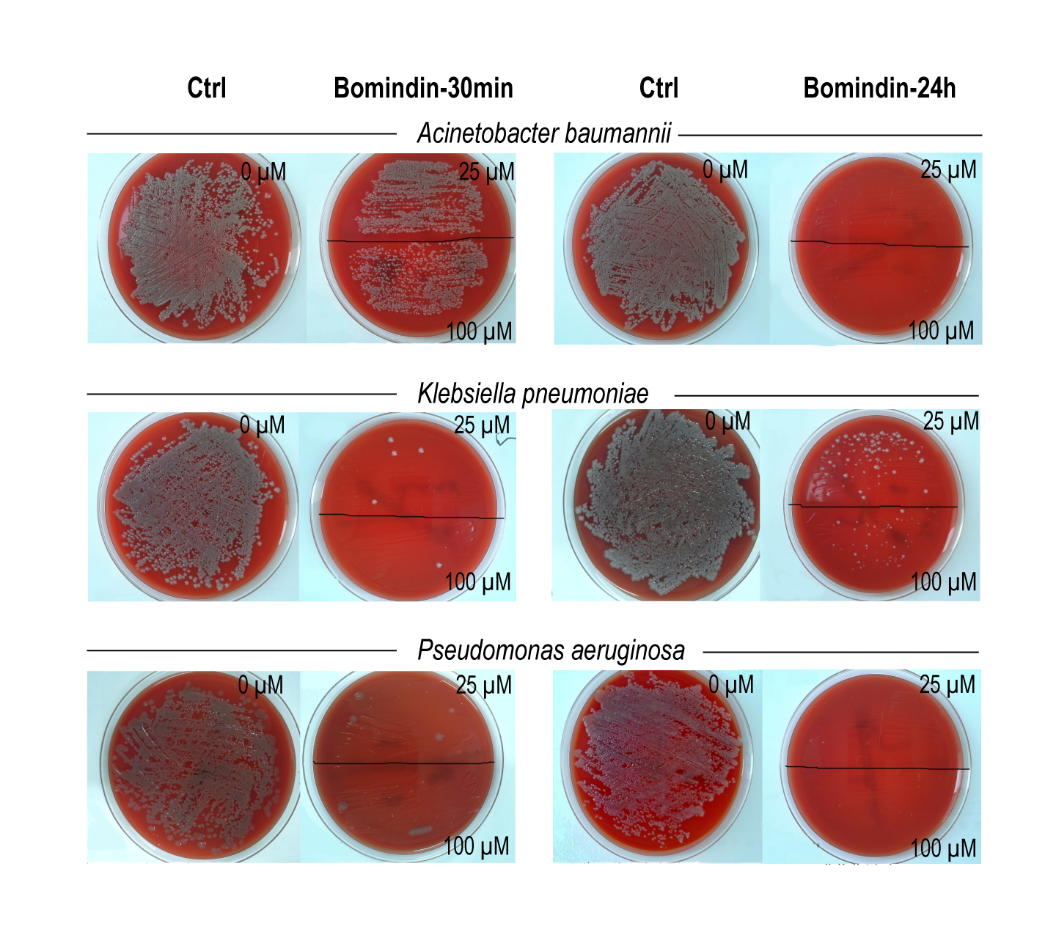
Supplementary Figures

**Figure S1.** Treatment significantly reduced bacteria populations. The 100 μM dose of bomidin abolished the growth of Klebsiella and Pseudomonas aeruginosa within 30 minutes, while the cultures of Acinetobacter baumannii showed inhibited growth within 24 h


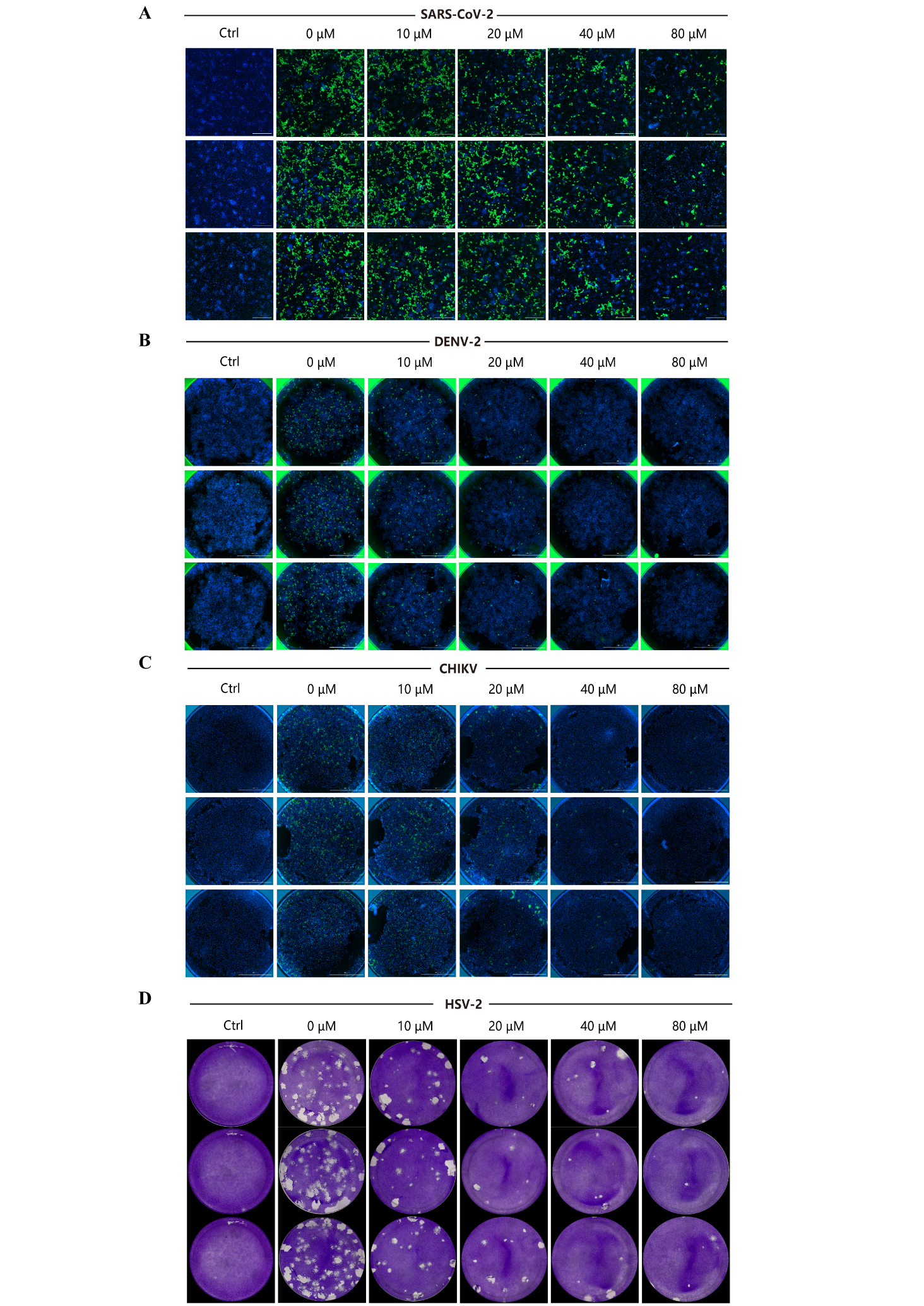


**Figure S2.** Broad spectrum antiviral activity of bomidin against viral infection in cells. Dose-dependent inhibitory effect of bomidin at different concentrations (0 μM, 10 μM, 20 μM, 40 μM and 80 μM) was determined by an immunofluorescence assay. **(A)** SARS-Cov-2, **(B)** DENV-2 and **(C)** CHIKV and  **(D)** the plaque forming assay (HSV-2) 48 h post-infection. Cell nuclei were stained with DAPI (blue). The viral proteins are indicated in green.


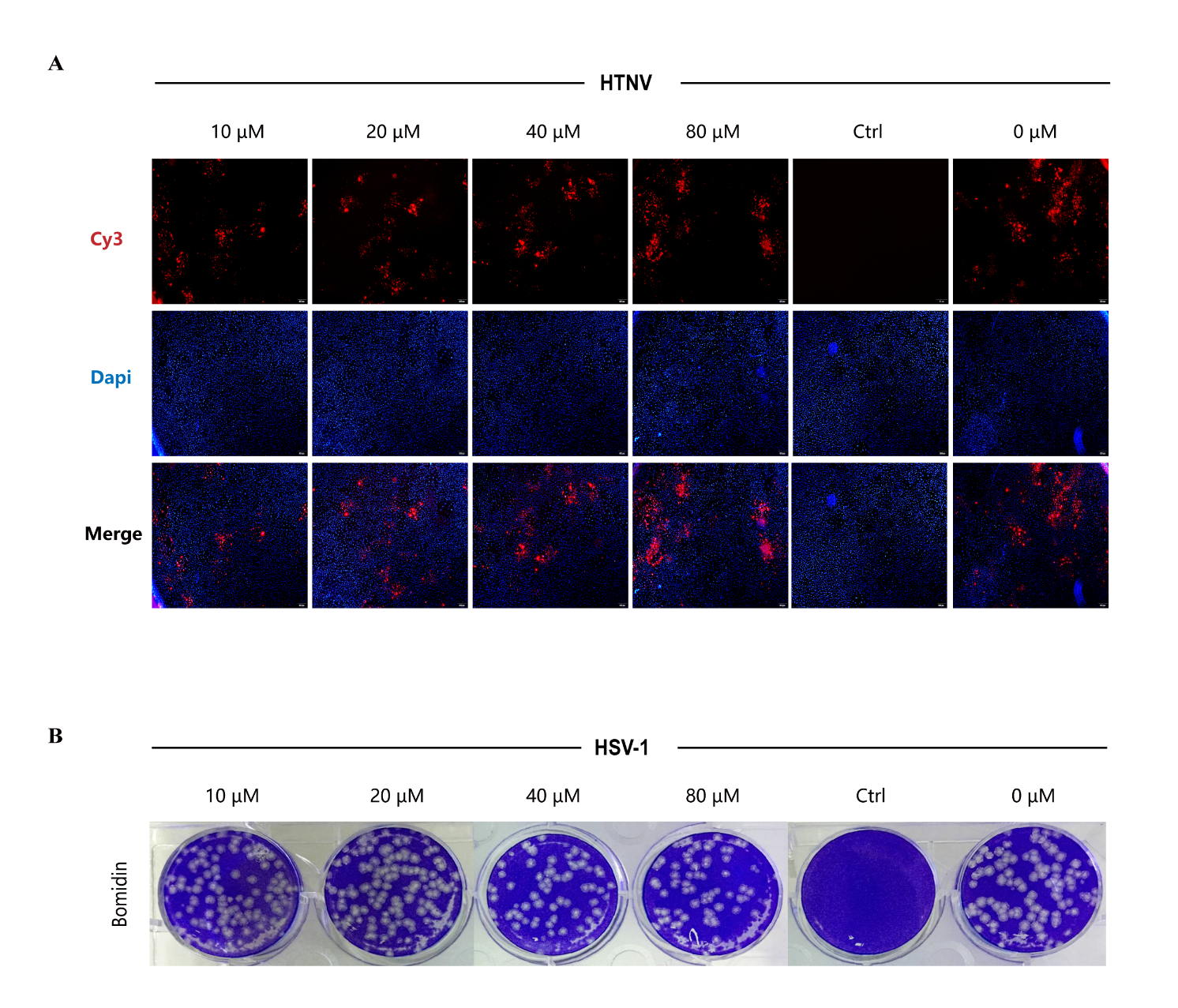


**Figure S3. (A)** Dose-dependent inhibitory effect of bomidin at different concentrations (0 μM, 10 μM, 20 μM, 40 μM and 80 μM) was determined by an immunofluorescence assay (HTNV).**（B）** the plaque forming assay (HSV-1) 48 h post-infection. Cell nuclei were stained with DAPI (blue). The viral proteins are indicated in green.

## Supplementary Videos


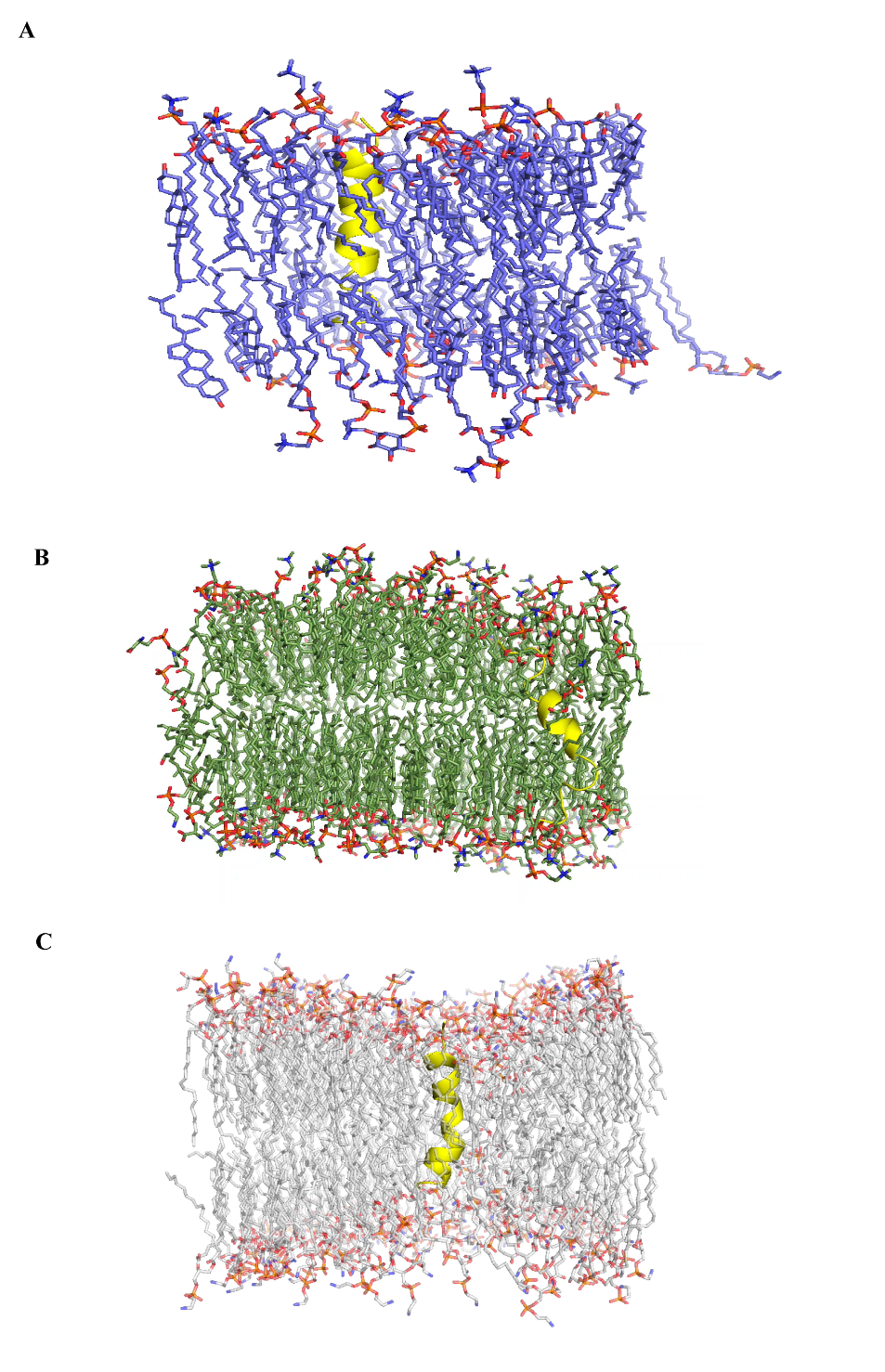


**Video S1.** Bomidin disrupted the surrounding lipid molecules and emerged from one side of the bilayer or one side of the membrane, possibly leading to the perforations or vesicular structures observed in the electron microscopy images. In contrast, bomidin resided in the plasma membrane without disrupting its overall integrity.


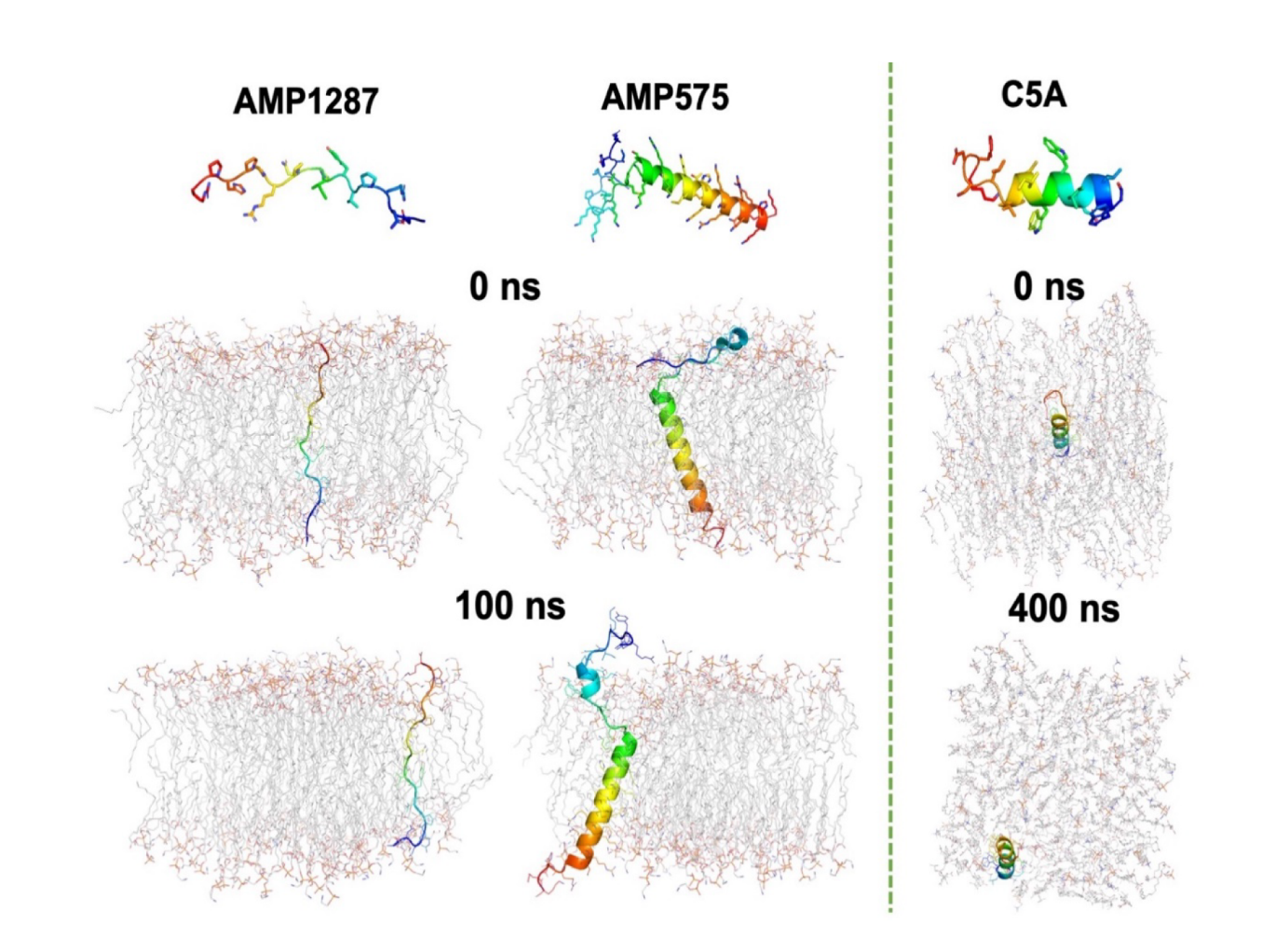


**Video S2**. Molecular dynamics simulations of there peptides with expeirmentally measured antimicrobial activities. These peptides featured membrane-disruption behaviors comparable to that of Bomidin.

The peptide sequences were adapted from the DBAASP database (https://dbaasp.org/). AMP1287: GNNRPVYIPQPRPPHPR

AMP575: GRYIAKINPDNKKFKTMPSGKKRKGHKMATHKRKKRLRKNRHKK

NS5A-derived peptide C5A: SWLRDIWDWICEVLSDFK

AMP1287 and AMP575 featured anti-bacterial activities, while NS5A-derived peptide C5A are anti-viral (HCV).


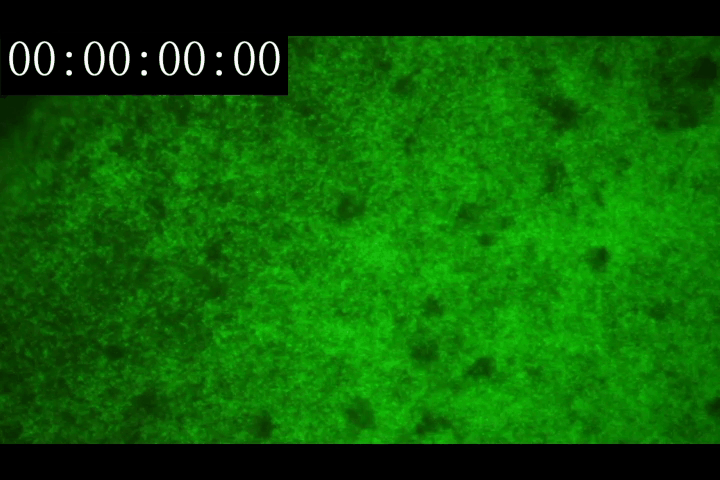


**Video S3.** After treatment with bomindin, the labeled fluorescent bacteria ruptured

## Supplementary Table.

## Table S1. Molar ratios of lipid components in computational membrane models.

|  | Viral  Membrane^*^ | Bacterial  Membrane^**^ | Human Plasma Membrane |
| --- | --- | --- | --- |
| PC phosphatidylcholine | **50** |  | 40 |
| PE phosphatidylethanolamine | 20 | **82** | 20 |
| PG phosphatidylglycerol |  | 6 |  |
| PS phosphatidylserine | 5 |  | 10 |
| PI phosphatidylinositol | 10 |  | 10 |
| CL cardiolipin |  | 12 |  |
| SM (sphingo) | 5 |  | **40** |
| Sterols | 10 |  | **40** |

* In current study, the components of viral membrane were adapted from shared lipidome of Dengue virus, SARS-CoV-2, HSV-2, and the host cell endoplasmic reticulum.

** E.Coli cell membrane was used as the model system, which are different from outer membrane of Gram-negative bacteria.
